# Protective effects and mechanisms of *Terminalia catappa* L. methenolic extract on hydrogen-peroxide-induced oxidative stress in human skin fibroblasts

**DOI:** 10.1186/s12906-018-2308-4

**Published:** 2018-10-01

**Authors:** Ya-Han Huang, Po-Yuan Wu, Kuo-Ching Wen, Chien-Yih Lin, Hsiu-Mei Chiang

**Affiliations:** 1 Department of Cosmeceutics, China Medical University, 91 Hsueh-Shih Road, Taichung, 40402 Taiwan; 2 Department of Dermatology, China Medical University Hospital, Taichung, 40402 Taiwan; 3 School of Medicine, China Medical University, 91 Hsueh-Shih Road, Taichung, 40402 Taiwan; 4 Department of Biotechnology, Asia University, 500 Liufeng Road, Wufeng District, Taichung City, 41354 Taiwan

**Keywords:** Oxidative stress, Reactive oxygen species, Aging, Hemeoxygenase-1 (HO-1), Extracellular matrix

## Abstract

**Background:**

Oxidative stress plays a crucial role in aging-related phenomenon, including skin aging and photoaging. This study investigated the protective role and possible mechanism of *Terminalia catappa* L. methanolic extract (TCE) in human fibroblasts (Hs68) against hydrogen peroxide (H_2_O_2_)-induced oxidative damage.

**Methods:**

Various in vitro antioxidant assays were performed in this study. The effect and mechanisms of TCE on oxidative stress-induced oxidative damage were studied by using western blotting.

**Results:**

The IC_50_ of TCE was 8.2 μg/mL for 1,1-diphenyl-2-picrylhydrazyl radical scavenging, 20.7 μg/mL for superoxide anion radical scavenging, 173.0 μg/mL for H_2_O_2_ scavenging, 44.8 μg/mL for hydroxyl radical scavenging, and 427.6 μg/mL for ferrous chelation activities. Moreover, TCE inhibited the H_2_O_2_-induced mitogen-activated protein kinase signaling pathway, resulting in the inhibition of c-Jun, c-Fos, matrix metalloproteinase (MMP)-1, MMP-3, MMP-9, and cyclooxygenase-2 expression. TCE also increased hemeoxygenase-1 expression inhibited by H_2_O_2_. Finally, TCE was demonstrated reverse type I procollagen expression in fibroblasts after H_2_O_2_ treatment.

**Conclusions:**

According to our findings, TCE is a potent antioxidant and protective agent that can be used in antioxidative stress-induced skin aging.

**Electronic supplementary material:**

The online version of this article (10.1186/s12906-018-2308-4) contains supplementary material, which is available to authorized users.

## Background

Aging can be divided into two basic processes: intrinsic aging, which is related to age, and extrinsic aging, which is generally due to long-term exposure to environmental factors, including ultraviolet (UV) light and pollutants. Oxidative stress plays a crucial role in aging-related disorders, including atherosclerosis, cardiovascular diseases and skin aging [1]. High levels of reactive oxygen species (ROS), such as hydrogen peroxide (H_2_O_2_), superoxide anion, and singlet oxygen, can cause oxidative damage to cellular DNA, protein, and lipids, resulting in the initiation or development of various disorders and diseases such as cardiovascular diseases, type 2 diabetes mellitus, and cancer [2]. In addition, free transition metal ions combine with H_2_O_2_ and can cause extensive oxidative damage to biomolecules such as lipids, proteins, and nucleic acids, leading to age-related disorders [3].

Skin aging is characterized by a sagging appearance, wrinkles, and pigmentary changes, and principally manifests as the degradation of extracellular matrix (ECM) proteins, including type I collagen, elastin, proteoglycans, and fibronectin [4, 5]. Type I collagen is the most abundant structural protein in skin connective tissue and is primarily synthesized by fibroblasts, whereas collagen in the dermis is responsible for skin strength and resiliency [6, 7]. Oxidative stress or inflammation can cause collagen degradation resulting in wrinkle formation and sagging skin [8]. In addition, ROS activate the mitogen-activated protein kinase (MAPK) pathway, which subsequently induces the expression and activation of matrix metalloproteinases (MMPs) in human skin [9]. The activation of MAPK and MMPs may cause damage and aging of the skin [10, 11]. Agents that can elevate ECM protein levels or downregulate collagen-degrading enzymes, such as MMPs, may prove useful in the development of effective antiaging agents [12, 13].

*Terminalia catappa* L. belongs to the family Combretaceae, and in Southeast Asia, it is commonly used as a folk medicine for treating hepatoma and hepatitis [14, 15]. The leaf and bark extracts of *T. catappa* have been reported to exhibit chemopreventive, antioxidant, hepatoprotective, and anti-inflammatory activities [16, 17]. *T. catappa* includes the phytochemicals of flavanoids (rutin, isoorientin, vitexin, and isovitexin), tannins (chebulagic acid, punicalagin, punicalin, and terflavins A and B), and triterpenoids (asiatic acid and ursolic acid) [14, 18]. In addition, the *T. catappa* extract exhibits antifungal and antidepressant activities [19, 20]. Topical application of ointment containing *T. catappa* was shown to promote wound healing in rats [21], and our previous study demonstrated that the *T. catappa* L. hydrophilic extract exerts protective effects on UVB-induced photoaging by inhibiting MMPs expression and upregulating type I procollagen expression [22]. However, the activity and related mechanisms of *T. catappa* against oxidative stress-induced skin damaging are unclear. Therefore, this study investigated the effects of *T. catappa* methanolic extract (TCE) on H_2_O_2_-induced skin damage and on the protein expression of MAPKs, which activate protein-1 (AP-1), MMPs, and type I procollagen in human skin fibroblasts (Hs68).

## Methods

### Chemicals

Fetal bovine serum (FBS), penicillin-streptomycin, trypsin-EDTA, and Dulbecco’s Modified Eagle’s Medium (DMEM) were purchased from Gibco, Invitrogen (Carlsbad, CA, USA). The Bradford reagent was supplied by Bio-Rad Laboratories (Hercules, CA, USA), and Tris and MTT were purchased from USB (Cleveland, OH, USA). Methanol, dimethyl sulfoxide, doxycycline hyclate, calcium chloride (CaCl_2_), DPPH, DL-dithiothreitol, and all other reagents used in this study were purchased from Sigma-Aldrich Chemicals (St. Louis, MO, USA).

### Preparation and quantitation of TCE

*T. catappa* leaves were collected in Wufeng, Taichung City, Taiwan, as previously described [22]. The leaves were identified by Professor KC Wen, a professor in Department of Cosmeceutics, China Medical University and a voucher specimen of this material (FCRDSAL-Plants-0003) has been deposited in Functional Cosmeceutics Research & Development and Safety Assessment Laboratory, China Medical University, Taiwan. The dried leaves (150 g) were ground and then extracted twice with 2 L of methanol for 1 h by using ultrasonication. The extraction liquid was filtrated, and the filtrate was evaporated to dryness in a vacuum to obtain TCE.

The total phenolic content of TCE was measured using the Folin–Ciocalteu reaction, as previously described [23]. Briefly, TCE was mixed with the Folin–Ciocalteu phenol reagent and sodium carbonate, and absorbance was measured at 760 nm. The phenolic content is expressed as microgram GAE/microgram *T. catappa* leaf dry weight herein.

The total flavonoid content of TCE was determined using the aluminum chloride colorimetric assay, as described elsewhere [23]. Briefly, TCE was mixed with aluminum chloride hexahydrate, potassium acetate, and deionized water, and the absorbance of the mixture was measured at 405 nm on an enzyme-linked immunosorbent assay (ELISA) reader (Tecan, Grödig, Austria). The flavonoid content is expressed as microgram QE/microgram *T. catappa* leaf dry weight herein.

### DPPH radical scavenging activity assay

DPPH was mixed with various concentrations of TCE. The mixture was added to a 96-well microplate and incubated at room temperature for 30 min in the dark. Subsequently, absorbance was measured at 492 nm on the ELISA reader. Ascorbic acid was used as a positive control [24, 25].

### Superoxide anion radical scavenging activity assay

Dihydronicotinamide-adenine dinucleotide, phenazinemethosulfate, and nitroblue tetrazolium were prepared in 0.1 M phosphate buffered saline (PBS), after which TCE was added. Absorbance was measured at 560 nm on the ELISA reader.

### Determination of peroxide scavenging activity

The peroxide scavenging activity of TCE was spectrophotometrically detected using a previously described method [23, 26]. H_2_O_2_ was prepared in PBS and mixed with various concentrations of TCE. Then, after incubation, absorption was measured at 230 nm on the ELISA reader.

### Hydroxyl radical scavenging activity assay

The hydroxyl radical scavenging activity assay was performed by mixing TCE, ascorbic acid, deoxyribose, iron (III) chloride, EDTA, H_2_O_2_, a monopotassium phosphate–potassium hydroxide buffer, and distilled water; the mixture was then incubated at 100 °C for 15 min and centrifuged. The absorbance of the supernatant was subsequently measured at 532 nm on a microplate reader (BioTek, Winooski, VT, USA). Mannitol was used as a positive control, and the hydroxyl radical scavenging activity of TCE was obtained as the percentage inhibition of deoxyribose degradation [3, 27].

### Ferrous ion chelating activity assay

Various concentrations of TCE were mixed with an iron (II) chloride solution. The reaction was initiated after ferrozine was added. Absorbance was then spectrophotometrically measured at 562 nm on the microplate reader. The results are expressed as the percentage inhibition of the generation of the ferrozine–ferrous complex herein [24].

### Measurement of reducing power

The reducing power of TCE was determined using a previously described method [24, 28]. Various concentrations of TCE were mixed with ferrocyanate and trichloroacetic acid. After centrifugation, the supernatant was mixed with ferric chloride and absorbance was measured at 700 nm. Ascorbic acid and distilled water were used as the positive and negative controls, respectively.

### Cell cultures

Hs68, HaCaT cells, and B16F0 cells were purchased from the Bioresource Collection and Research Center in Hsinchu, Taiwan. These cells were maintained in DMEM containing 10% FBS, 100 U/mL penicillin, and 100 U/mL streptomycin in an incubator set at 37 °C.

### Cell viability assay for three skin cell lines

To understand the cytotoxicity of TCE on the skin, Hs68, HaCaT cells, and B16F0 cells were applied to study the cell viability. The cells were seeded in the plate, allowed to attach overnight, and were treated with 1 mL of various concentrations of TCE dissolved in DMEM for 24 h. The cytotoxicity of TCE was then evaluated using the MTT assay, as described elsewhere [22].

### Fluorescence assay for IntracellularROS generation in fibroblasts

Intracellular ROS generation was measured using a previously detailed method [22]. In brief, fibroblasts were added to a 24-well plate and then incubated with various concentrations of TCE for 24 h. The cells were washed with PBS and incubated with 150 μM H_2_O_2_ for 1 h. Subsequently, the cells were incubated with 10 μM DCFDA in DMEM for 30 min, after which they were examined under a fluorescence microscope (Leica DMIL, Wetzlar, Germany). Fluorescence (emission wavelength: 520 nm; excitation wavelength: 488 nm) was measured on a microplate reader (Thermo Electron Corporation, Vantaa, Finland).

### Western blotting

The cells were incubated with TCE (5–50 μg/mL) for 4 h, followed by incubation with 150 μM H_2_O_2_ for 1 h. The cells were collected and lysed with protein extraction buffer, as previously described [22]. An equal amount of protein was loaded, separated on 10% sodium dodecyl sulfate polyacrylamide gels, and then electrophoretically transferred to a polyvinylidene difluoride membrane. The membrane was incubated with specific antibodies against MMP-1, − 3, and − 9; type I procollagen; HO-1; MAPKs; c-Jun; c-Fos; and COX-2 (Santa Cruz Biotechnology, Inc., Santa Cruz, CA, USA). The blots were then incubated with anti-immunoglobulin G-horseradish peroxidase and chemiluminescent detection reagent (Amersham Biosciences, Buckinghamshire, United Kingdom). Finally, immunoreactive bands were detected using a chemiluminescent detection system (LAS-4000, Fujifilm, Tokyo, Japan), and the density of the bands was determined using a densitometric program (Multi Gauge V2.2, Fujifilm, Tokyo, Japan).

### Statistical analyses

Values are presented as the mean ± standard deviation of at least three independent experiments. The results were analyzed using one-way analysis of variance, followed by Scheffe’s test. Statistical significance was set at *p* < 0.05.

## Results

### Extraction yield and quantitation of TCE

The extraction yield of TCE from leaves was 11.5%. The total phenolic content of the extract was determined using the Folin–Ciocalteu method, and the regression coefficient of the calibration curve was 0.9995. Specifically, the total phenolic content of TCE was 220.2 ± 0.2 μg/mg gallic acid equivalent (GAE). Additionally, the total flavonoid content of TCE was determined using the aluminum chloride colorimetric method, and the regression coefficient of the calibration curve was 0.9991. The total flavonoid content was 109.0 ± 0.8 μg/mg quercetin equivalent (QE). The content of gallic acid was 74.62 μg/mL by HPLC/UV analysis (Additional file 1: S1).

### The antioxidant activity of TCE

**Fig. 1:**
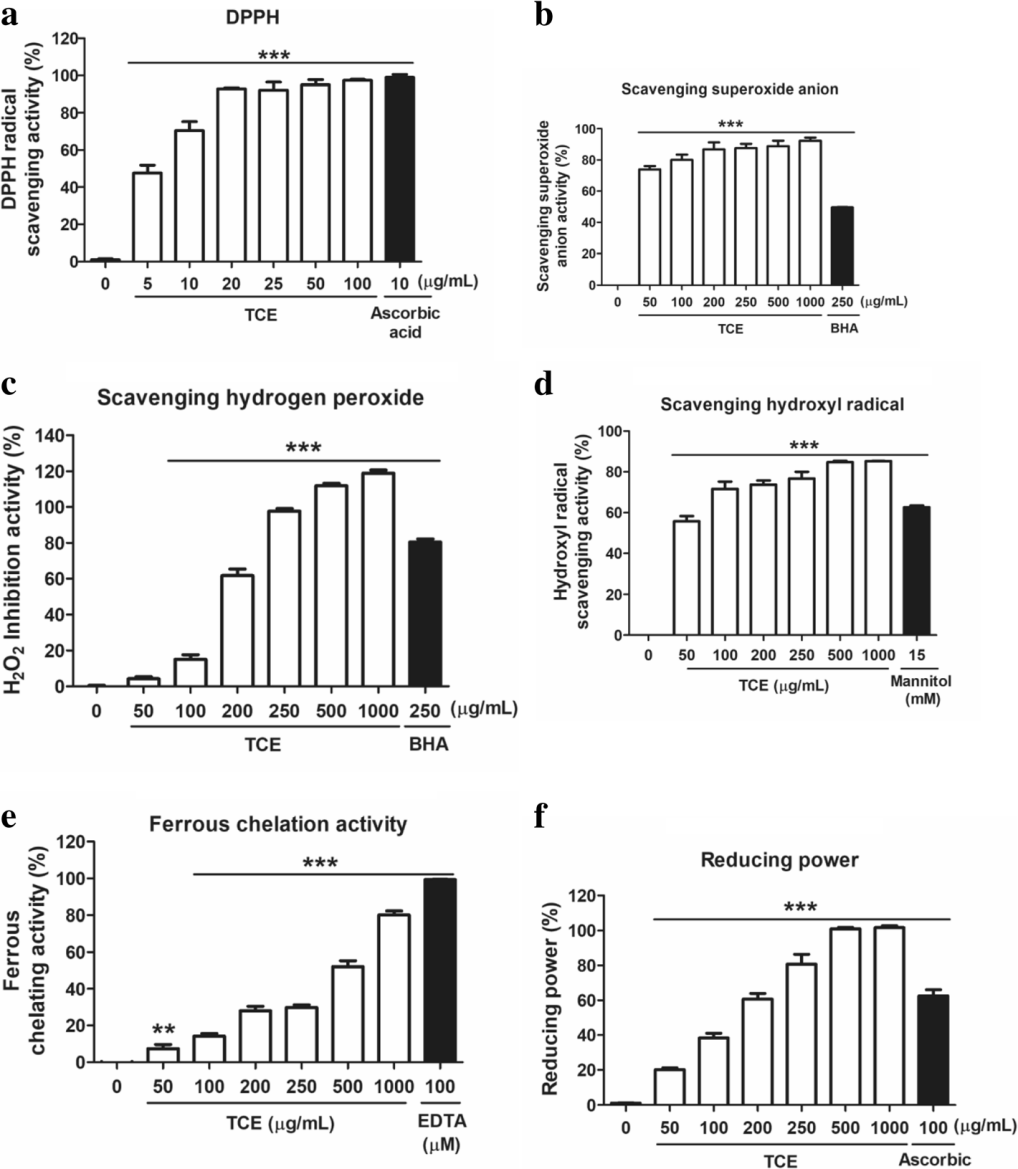
The aioxidant activity of *Terminalia catappa* L. methenolic extract (TCE). **a** 1,1-diphenyl-2-picrylhydrazyl radical scavenging activity of TCE; **b** Superoxide anion radical scavenging activity of TCE; **c** Peroxide scavenging activity of TCE; **d** Hydroxyl radical scavenging activity of TCE; **e** Ferrous chelating activity of TCE; and **f** Reducing power of TCE. Significant difference versus control (without extract): *p < 0.05; **p < 0.01; ***p < 0.001

The antioxidant activity of TCE was study by using free radical scavenging assay and chelating assay. Figure 1a shows the DPPH radical scavenging activity of TCE and 10 μg/mL ascorbic acid (positive control). The results indicated that 10 μg/mL TCE exhibited a scavenging activity of 70.4% ± 4.9%, and that the activity was 99.0% ± 1.6% for the same concentration of ascorbic acid. The IC_50_ of TCE for DPPH scavenging activity was 5.6 μg/mL; in other words, TCE preparations exhibited potent DPPH free radical scavenging activity. As shown in Fig. 1b, the superoxide anion radical scavenging activity was 49.5% ± 0.2% for 250 μg/mL beta hydroxyl acid (BHA) (positive control), and ranged from 73.9% ± 2.1% to 92.4% ± 2.0% for 50–1000 μg/mL TCE. The IC_50_ of TCE for superoxide anion radical scavenging was 20.6 μg/mL. Thus, the superoxide anion radical scavenging activity of TCE was superior to that of BHA. The peroxide scavenging activities of TCE (50–1000 μg/mL) and the positive control BHA (250 μg/mL) are shown in Fig. 1c. Specifically, the peroxide scavenging activity ranged from 4.2% ± 1.2% to 111.8% ± 1.3% for various concentrations of TCE, and was 80.4% ± 1.8% for BHA. Notably, the peroxide scavenging activity of TCE was superior to that of BHA (IC_50_ = 166.1 μg/mL). The hydroxyl radical scavenging activities of TCE (50–1000 μg/mL) and the positive control mannitol (15 mM) are shown in Fig. 1d. Specifically, the hydroxyl radical scavenging activity ranged from 55.7% ± 2.6% to 85.3% ± 0.1% for various concentrations of TCE, and was 62.0% ± 0.9% for mannitol. The IC_50_ of TCE for hydroxyl radical scavenging was 39.6 μg/mL.

Figure 1e shows the metal chelating activities of TCE and the positive control ethylenediaminetetraacetic acid (EDTA). The activities ranged from 7.5% ± 2.1% to 80.0% ± 2.3% for various concentrations of TCE (50–1000 μg/mL), and was 99.4% ± 0.1% for EDTA (100 μM). The IC_50_ of TCE was 427.6 μg/mL for metal chelation. The reducing power ranged from 20.1% ± 1.0% to 101.7% ± 1.2% for 50–1000 μg/mL TCE, whereas the reducing power for 100 μg/mL ascorbic acid (positive control) was 62.6% ± 3.5% (Fig. 1f). The IC_50_ of TCE was 128.5 μg/mL.

### TCE inhibited H_2_O_2_^−^Induced cytotoxicity and intracellular ROS generation

**Fig. 2:**
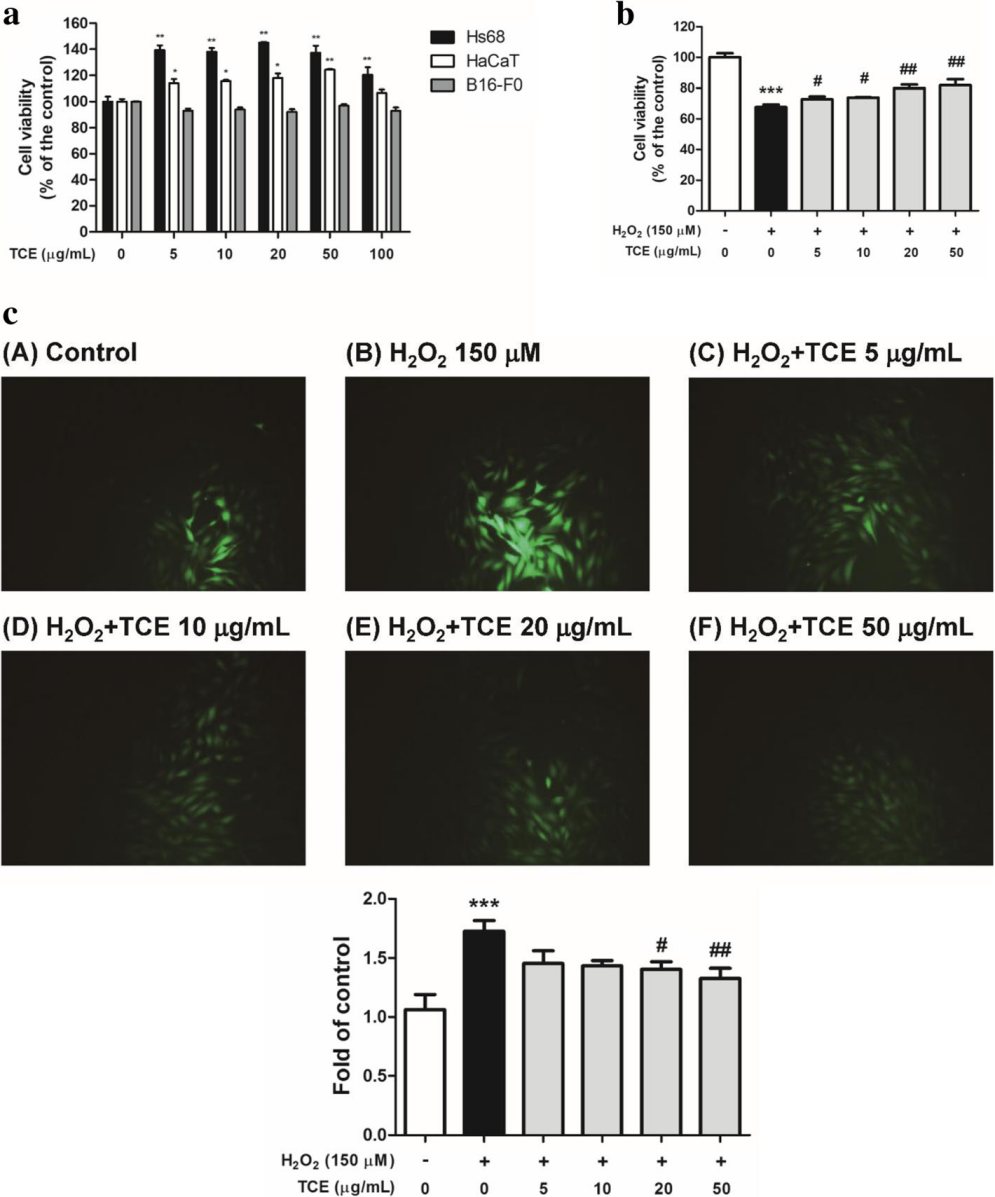
The cytotoxicity and effect on intracellular oxidative stress in Hs68 of TCE. **a** Cell viability (%) of human fibroblasts (Hs68), human keratinocytes, and mouse melanoma cells treated with TCE; **b** Cell viability of Hs68 after treatment with TCE with or without 150 μM hydrogen peroxide (H_2_O_2_) exposure. **c** Repressive effect of TCE on H_2_O_2_-induced intracellular oxidative stress in Hs68. Significant difference versus control: *p < 0.05; **p < 0.01; ***p < 0.001. Significant inhibition versus H_2_O_2_-exposed group: ^#^*p* < 0.05; ^##^*p* < 0.01

Human fibroblasts (Hs68), human keratinocytes (HaCaT), and mouse melanoma cells (B16F0) were treated with various concentrations of TCE (5–100 μg/mL), and their cell viability was measured using the 3-(4,5-dimethylthiazol-2-yl)-2,5-diphenyltetrazolium bromide (MTT) assay. As shown in Fig. 2a, the results indicated that TCE did not exhibit cytotoxic effects in the three skin cell lines; these concentrations were thus applied in subsequent experiments.

As shown in Fig. 2b, cell viability was 67.6% ± 1.7% after H_2_O_2_ treatment. Cell viability ranged from 72.7% ± 1.8% to 81.9% ± 3.9% for 5–50 μg/mL TCE. These results indicated that TCE protects the skin from oxidative stress-induced cytotoxicity.

The 2′,7′-dichlorofluorescin diacetate (DCFDA) fluorescence assay was used to qualitatively characterize intracellular ROS generation. As shown in Fig. 2c, ROS levels were markedly higher in H_2_O_2_-exposed fibroblasts than in control cells. Moreover, this increase in ROS generation was attenuated in H_2_O_2_-exposed fibroblasts pretreated with various concentrations of TCE (5–50 μg/mL). ROS generation in H_2_O_2_-exposed fibroblasts increased to 1.7-fold compared with control cells, and significantly decreased to 1.3-fold compared with control cells. TCE at 50 μg/mL decreased H_2_O_2_-induced intracellular ROS generation by 23.1%. Thus, TCE protects the skin from ROS damage.

### Inhibition of MAPK phosphorylation through TCE

**Fig. 3:**
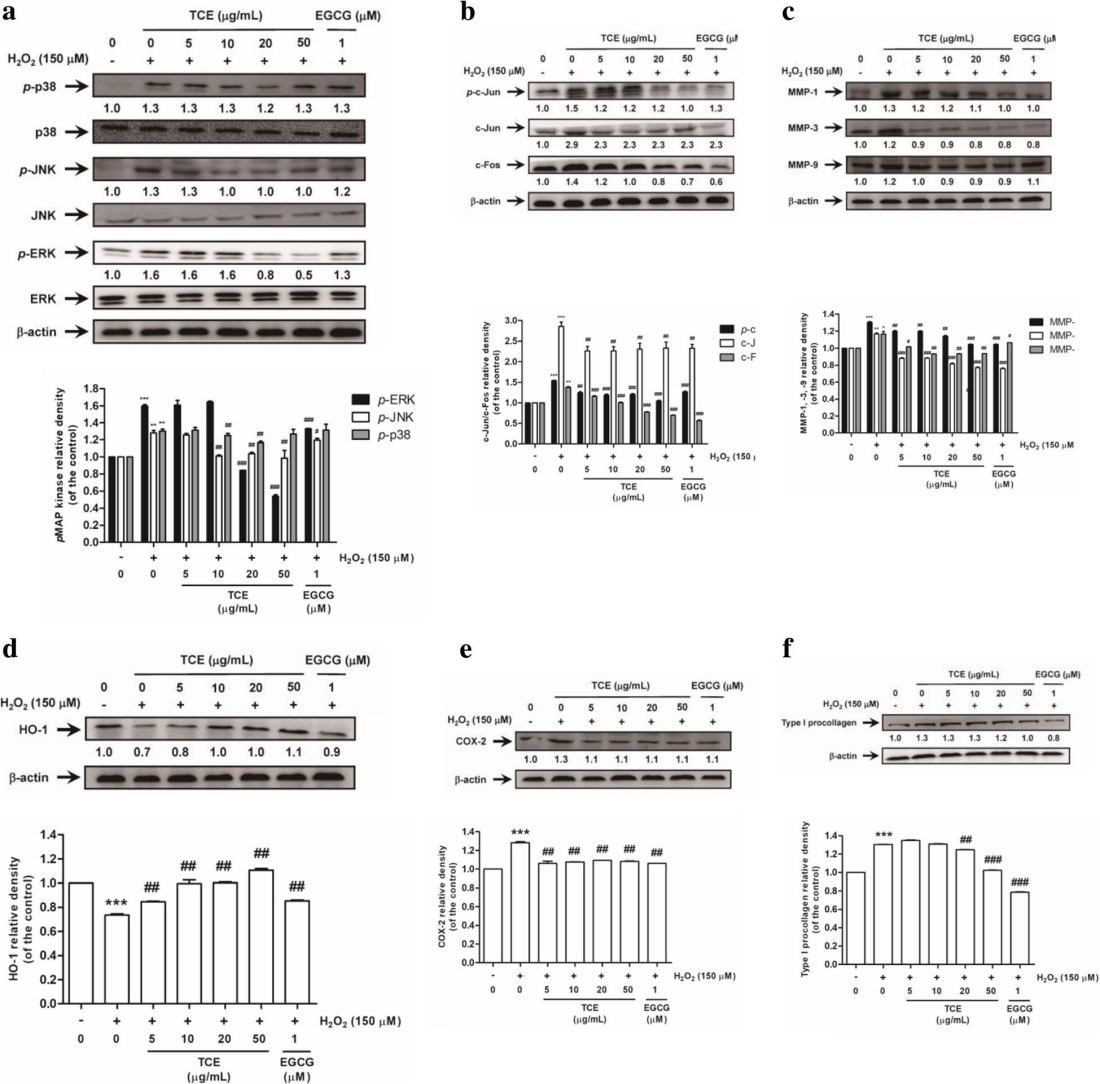
Effect of TCE on the H_2_O_2_-induced (**a**) phosphorylation of mitogen-activated protein kinases, (**b**) c-Jun phosphorylation and c-Fos, (**c**) matrix metalloproteinase (MMP)-1, − 3, − 9, (**d**) hemeoxygenase-1 (HO-1), (**e**) cyclooxygenase-2 (COX-2) and (**f**) type I procollagen protein expressions in Hs68. Significant difference versus control: *p < 0.05; **p < 0.01; ***p < 0.001. Significant inhibition versus H_2_O_2_-exposed group: ^#^*p* < 0.05; ^##^*p* < 0.01; ^###^*p* < 0.001. EGCG: (−)-epigallocatechin gallate was used as a positive control

As shown in Fig. 3a, H_2_O_2_ induced the phosphorylation of p38, extracellular signal–regulated kinase (ERK), and c-Jun N-terminal kinase (JNK). TCE (5–50 μg/mL) dose-dependently inhibited the phosphorylation of ERK, and the effect was significant in the cells treated with > 20 μg/mL TCE. Similar to the effect on ERK, TCE inhibited JNK and p38 activation, which were significantly suppressed when TCE concentration was 20 μg/mL.

### TCE inhibited phosphorylation of AP-1 in Hs68

As shown in Fig. 3b, H_2_O_2_ increased c-Jun and *p*-c-Jun expression to 1.5- and 2.9-fold that of the control, respectively, whereas > 5 μg/mL TCE significantly reduced the effect. In addition, H_2_O_2_ induced c-Fos expression, but TCE reduced the effect. These results further indicated that TCE protects the skin from oxidative stress-induced damage.

### Effect of TCE on MMP expression in Hs68

To examine whether TCE protects H_2_O_2_-exposed Hs68 from oxidative stress-induced damage, the expression of cellular MMP-1, − 3, and − 9 proteins was measured. As depicted in Fig. 3c, H_2_O_2_ significantly elevated the expression of MMP-1, − 3, and − 9 proteins by 1.3-, 1.2-, and 1.2-fold compared with controls in Hs68, respectively. By contrast, TCE attenuated H_2_O_2_-induced MMP expression. Specifically, treatment with > 5 μg/mL TCE significantly reduced H_2_O_2_-induced MMP-1, − 3, and − 9 expression. These results indicated that TCE prevents the H_2_O_2_-induced elevation of MMP-1, − 3 and − 9 levels, thus protecting the skin from oxidative stress-induced damage.

### Effect of TCE on H_2_O_2_-induced Hemeoxygenase-1 expression

The hemeoxygenase (HO)-1 gene and protein play a pivotal role in the modulation of antioxidant, anti-inflammatory, and antiapoptotic activities. This study revealed that H_2_O_2_ significantly reduces HO-1 protein expression in Hs68, whereas TCE treatment dose-dependently increases HO-1 expression (Fig. 3d).

### Effect of TCE on H_2_O_2_-induced Cyclooxygenase-2 expression in Hs68

Cyclooxygenase (COX)-2 levels were 1.3-fold higher in fibroblasts exposed to 150 μM H_2_O_2_ than in control cells (Fig. 3e). In addition, various concentrations of TCE (5–50 μg/mL) reduced COX-2 expression; the effect was significant in the cells treated with > 5 μg/mL TCE. These results further confirmed that TCE protects the skin from damage by inhibiting inflammation.

### Reversal of H_2_O_2_-induced upregulation of type I procollagen expression in Hs68 through TCE

After treatment with 150 μM H_2_O_2_, the expression of type I procollagen increased to 1.3-fold compared with that in control cells, whereas TCE inhibited this effect (Fig. 3f). Notably, treatment of the cells with 50 μg/mL TCE decreased type I procollagen expression to a level similarly expressed in the control cells.

## Discussion

Polyphenols are the second most abundant metabolic products in plants. Notably, plants with high polyphenolic content exhibit potent antioxidant activity [29]. Free radical scavenging activity is related to the polyphenic and flavonoid content of plants. In a previous study, the total phenol content of *Rosa hemisphaerica* was 138.3 μg/mg GAE [30]. In the present study, the total phenolic content was 220.2 μg/mg GAE dry leaves, the total flavonoid content was 109.0 μg/mg QE dry leaves, and the IC_50_ of TCE for DPPH radical scavenging was 5.6 μg/mL. In addition, TCE exhibited strong scavenging activity for ROS including superoxide, peroxide, and hydroxyl radicals. Peroxide is the primary product of initial oxidation, and it can react with ferrous ions, producing more toxic hydroxyl radicals. Iron also has high reactivity and is the pivotal factor in lipid peroxidation catalyzed by transition metals [3]. Furthermore, TCE exhibits potent metal chelating activity and reducing power attenuating features; in the present study, TCE attenuated H_2_O_2_-induced metal chelation, reducing power, ROS generation, and free radical scavenging. Our results suggest that the high polyphenic and flavonoid content of TCE contribute to it potent antioxidant activity.

Molecules such as glutathione, catalase, and HO-1 provide cells, and the body overall, with defense systems against intrinsic and extrinsic oxidative stress. Nuclear factor E2-related factor 2 (Nrf2) and Keap1 are redox-sensitive transcription factors and key intracellular modulators of antioxidant defense against environmental stresses. For example, Nrf2 has been reported to protect skin cells from UV- and pollutant-induced oxidative damage and cellular dysfunction [31]. On exposure to oxidative stress, Nrf2 is translocated to the cell nucleus and binds to antioxidant elements, activating phase II detoxification enzymes such as HO-1 and glutathione [32]. In the present study, H_2_O_2_ was found to reduce HO-1 expression; however, TCE treatment increased HO-1 expression, alleviating H_2_O_2_-induced oxidative stress in the skin cells. In other words, TCE may repair or protect skin from the damage caused by superoxide peroxide and hydroxyl radicals.

Exposure of the skin to UV induces ROS generation and regulates the expression of genes and proteins, resulting in photodamage and photocarcinogenesis [7]. In addition, H_2_O_2_ has been reported to cause skin aging by inducing oxidative stress and MMP expression [33], while UVB-induced ROS generation triggers ERK, JNK, and p38 phosphorylation, AP-1 activation, and MMP expression, leading to collagen degradation [7]. In addition, H_2_O_2_ disrupts transforming growth factor beta transduction and subsequently inhibits collagen biosynthesis, inducing skin aging [8]. In the present study, H_2_O_2_ was determined to upregulate the phosphorylation of MAPKs, c-Jun, c-Fos, and MMP-1, − 3, and − 9 proteins, whereas TCE inhibited these effects. This finding suggests that TCE activity is dependent on this signaling transduction. MMPs mediate degradation of ECM and play an important role in tissue homeostasis and remodeling including angiogenesis and tissue repair. Over suppression of MMPs may cause abnormal accumulation of ECM.

The results are consistent with those of our previous study, in which the *T. catappa* water extract protected skin from photodamage by inhibiting the MAPK/AP-1/MMP pathway [22]. UVB rays inhibited collagen synthesis and induced collagen degradation, whereas *T. catappa* water extract elevated the collagen content in Hs68 [22]. Similarly, in the present study, H_2_O_2_ was found to increase the collagen content in Hs68, whereas TCE reversed the effect. One previous study showed that H_2_O_2_ also reduces mRNA expression of type I collagen (COL1A1) in fibroblasts [34], although these results are inconsistent with those reported elsewhere. For example, researchers demonstrated that H_2_O_2_ induces oxidative stress damage to cells and the body, which can trigger the repair process of the skin, thereby increasing the collagen content. However, excessive collagen synthesis may cause collagen fibrosis and scleroderma [35]. Overall, our results here indicate that TCE can regulate the collagen content within a normal range.

## Conclusion

**Fig. 4:**
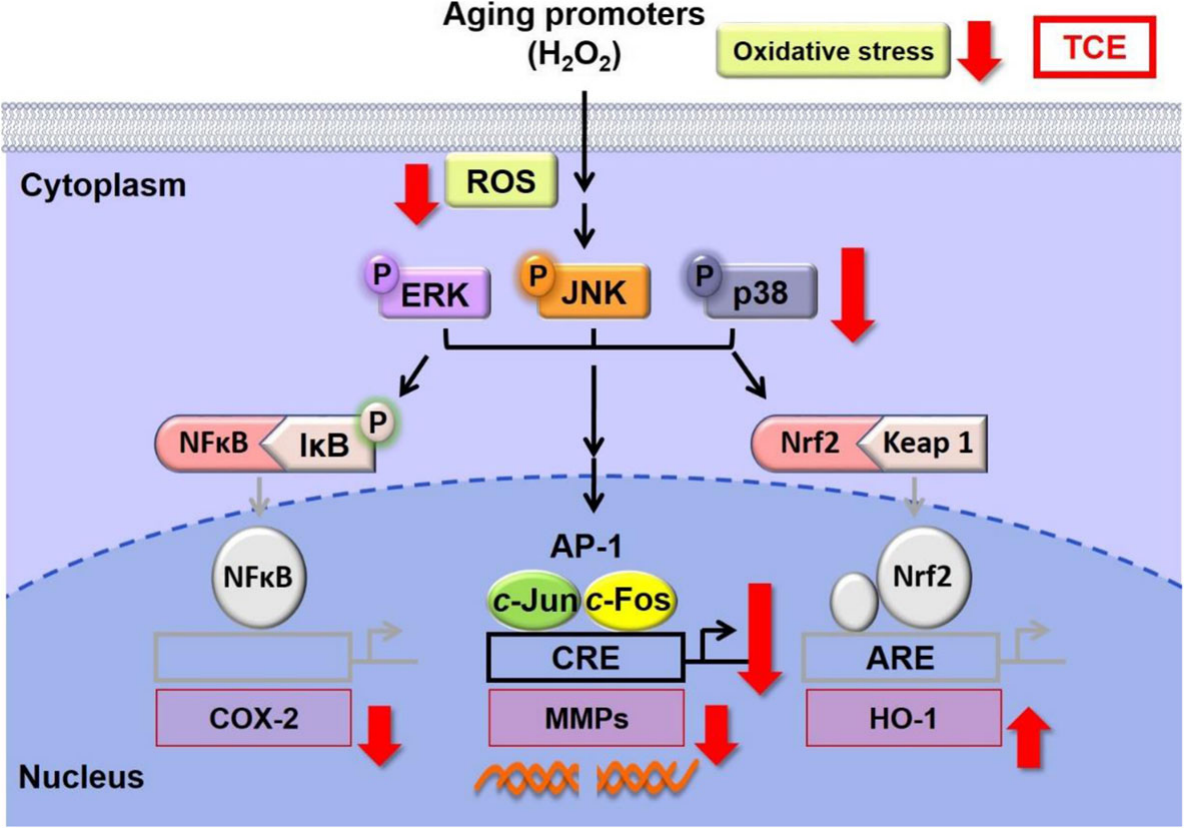
Scheme for TCE inhibition of oxidative stress-induced skin damage. (↑: upregulation; ↓inhibition)

The present study indicated that TCE with high polyphenic and flavonoid content exhibits potent free radical scavenging and antioxidant activity. Specifically, we determined that TCE protects against H_2_O_2_-induced skin damage by inhibiting the protein expression of MMP, AP-1, MAPKs, and COX-2 (Fig. 4). The antioxidant and antiaging activities of TCE make it suitable for application in skin care products.

## Additional file

Additional file 1: **S1.** The active components in *Terminalia catappa* L. methanolic extract (TCE). (DOCX 134 kb)

## Abbreviations

AP-1: Activate protein-1
BHA: Beta hydroxyl acid
COX: Cyclooxygenase
DCFDA: 2′,7′-dichlorofluorescin diacetate
DMEM: Dulbecco’s Modified Eagle’s Medium
ECM: Extracellular matrix
EDTA: Ethylenediaminetetraacetic acid
ELISA: Enzyme-linked immunosorbent assay
ERK: Extracellular signal–regulated kinase
FBS: Fetal bovine serum
GAE: Gallic acid equivalent
H_2_O_2_: Hydrogen peroxide
HO-1: Hemeoxygenase-1
JNK: C-Jun N-terminal kinase
MAPK: Mitogen-activated protein kinase
MMP: Matrix metalloproteinase
MTT: 3-(4,5-dimethylthiazol-2-yl)-2,5-diphenyltetrazolium bromide
Nrf2: Nuclear factor E2-related factor 2
PBS: Phosphate buffered saline
QE: Quercetin equivalent
ROS: Reactive oxygen species
TCE: *Terminalia catappa* L. methanolic extract
UV: Ultraviolet
