# Supplementary material for: Protective effects and mechanisms of Terminalia catappa L. methenolic extract on hydrogen-peroxide-induced oxidative stress in human skin fibroblasts
Source: BMC Complement Altern Med. 2018 Oct 1;18:266. doi: 10.1186/s12906-018-2308-4 (PMC6167875; doi:10.1186/s12906-018-2308-4)
Supplement: Supplementary file 1 — S1. The active components in Terminalia catappa L. methanolic extract (TCE). (DOCX 134 kb) [file 12906_2018_2308_MOESM1_ESM.docx]

**Addition file 1 S1**

**The active components in *Terminalia catappa* L. methanolic extract (TCE)**

**Instrument and Condition of HPLC analysis**

Acetonitrile, and glacial acetic acid were purchased from Avantor Chemicals. The HPLC apparatus was equipped with a pump (LC-20AT vp, Shimadzu, Japan), an automatic injector (SPD-20AF, Shimadzu, Japan), a UV-VIS detector (SPD20A vp, Shimadzu, Japan), and a Cosmosil 5C18 AR-II 5μ column (4.6 × 250 mm, Nacalai Tesque, Kyoto, Japan).

Among all tried experiments, the mobile phase combination is 0.5% acetic acid in water: acetonitrile (80:20%v/v). The solution was degassed in an ultrasonic water bath for 5 minutes and filtered through 0.45μm nylon filter. The detection of compounds of interest was performed at a wavelength (λ) of 254 nm, and the volume injected was 10 μl, with the mobile phase flow set at 1.0 mL/min.[[1](#_ENREF_1)]

**Results**

The content of gallic acid was 74.62 μg/mL by HPLC/UV analysis.

(a)


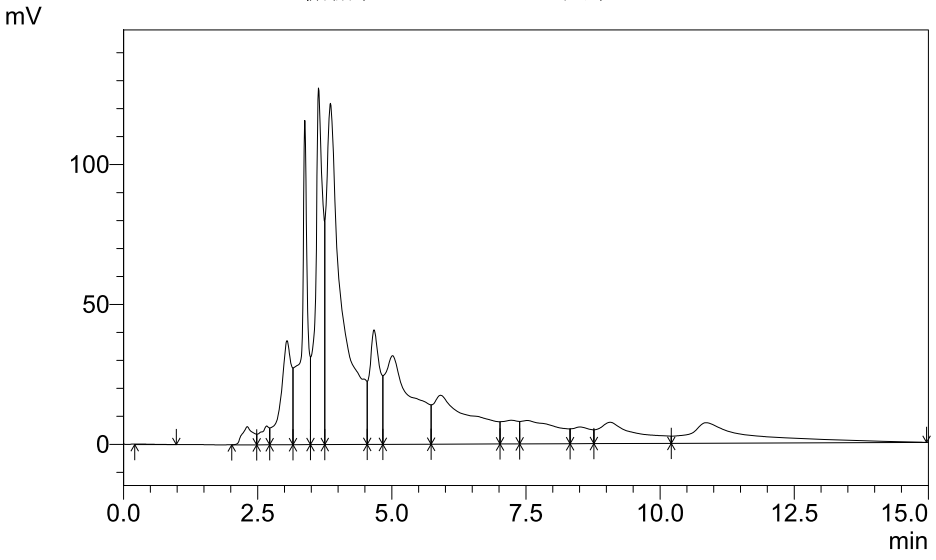


**(b)**

Figure. The HPLC chromatography of (a) TCE and (b) gallic acid.

**Reference**

1. Dhanani T, Shah S, Kumar S: **A validated high-performance liquid chromatography method for determination of tannin-related marker constituents gallic acid, corilagin, chebulagic acid, ellagic acid and chebulinic Acid in four Terminalia species from India**. *Journal of chromatographic science* 2015, **53**(4):625-632.
